# Tuberculosis of the Sphenoid Sinus Causing Blindness in a Pediatric Patient: A Case Report

**DOI:** 10.7759/cureus.116803

**Published:** 2026-09-23

**Authors:** Pradipt R Sahoo, Manas Ranjan Rout, Sreyash Chowdhary, Sharan Sinha, Utsav Singhvi

**Affiliations:** 1 Otolaryngology, Kalinga Institute of Medical Sciences, Bhubaneshwar, IND; 2 Otolaryngology - Head and Neck Surgery, Kalinga Institute of Medical Sciences, Bhubaneshwar, IND; 3 Otorhinolaryngology, Kalinga Institute of Medical Sciences, Bhubaneshwar, IND

**Keywords:** case report, extrapulmonary tuberculosis, pediatric tuberculosis, skull base, sphenoid sinus

## Abstract

Tuberculosis (TB) of the paranasal sinuses is an extremely rare manifestation of extrapulmonary disease, with the sphenoid sinus being the least frequently affected site. Due to its deep anatomical location and proximity to vital neurovascular structures, it often mimics malignancy or fungal infections, frequently leading to delayed diagnosis and severe neurological complications.

A 10-year-old male presented with a progressive generalized headache and severe vision loss in his left eye lasting two months. Ophthalmic examination confirmed left-sided optic nerve involvement. Advanced imaging revealed a large lytic mass centered in the sphenoid sinus with erosion of the sella turcica and clivus, bulging into the cavernous sinus and pituitary fossa. Initial endoscopic histopathology showed necrotic granulomatous inflammation but was negative for acid-fast bacilli (AFB). Following a postoperative seizure, endoscopic re-exploration was performed. Cartridge-based nucleic acid amplification testing (CBNAAT) of the tissue returned positive for *Mycobacterium tuberculosis*. The patient was initiated on standard four-drug antitubercular therapy (ATT), resulting in improvement in visual acuity to 6/6 at six-month follow-up.

Isolated sphenoid sinus tuberculosis is an elusive skull base lesion that demands high clinical suspicion. Early surgical intervention for decompression and tissue diagnosis, combined with prompt initiation of antitubercular therapy, is vital to prevent permanent neuro-ophthalmic deficits.

## Introduction

Tuberculosis (TB) remains a major global public health challenge and a leading infectious cause of morbidity and mortality worldwide, typically manifesting as pulmonary disease. However, extrapulmonary TB (EPTB) constitutes approximately 15% to 20% of all recorded TB cases, with its prevalence increasing further in pediatric and immunocompromised cohorts. Within EPTB, head and neck involvement accounts for roughly 10% of presentations, predominantly localized to the cervical lymph nodes.

Sinonasal TB is an extreme clinical rarity, representing less than 1% of all head and neck TB cases. When the paranasal sinuses are involved, the disease typically targets the highly vascularized mucosa of the maxillary and ethmoid sinuses. Isolated involvement of the sphenoid sinus is the rarest manifestation of all, accounting for a mere 1% to 2% of sinonasal TB cases. This rarity is primarily attributed to its deep-seated skull base anatomy, limited direct exposure to inhaled droplets, and sparse localized lymphatic drainage. Since the first historical documentation of primary sphenoid sinus TB over a century ago, the entity has remained an exceptional anomaly in modern rhinological and neurosurgical practice. The historical literature indicates that the first documentation of primary TB originating directly within the sphenoid sinus dates back over a century [1].

Managing sphenoid sinus TB presents extraordinary diagnostic hurdles. Located at the central skull base, the sphenoid sinus sits in immediate proximity to vital neurovascular structures, including the optic nerves and chiasm, the cavernous sinuses, the internal carotid arteries, and cranial nerves III, IV, V1, V2, and VI.

Because the sinus is deeply hidden, early localized symptoms are absent. Instead, patients present with vague, nonspecific complaints such as progressive, deep-seated headaches, visual disturbances, or cranial nerve palsies.

The primary diagnostic challenge lies in its ability to masquerade as more common, aggressive skull base lesions. Advanced imaging, including computed tomography (CT) and magnetic resonance imaging (MRI), frequently demonstrates extensive osteolytic bone destruction of the clivus and sella turcica, mimicking aggressive malignancies, chordomas, or invasive fungal sinusitis (e.g., mucormycosis). This subtle clinical picture leads to frequent misdiagnoses as fungal sinusitis, malignancy, or chronic inflammatory lesions [2,3]. Furthermore, establishing a microbiological diagnosis is highly difficult; tissue biopsies in EPTB are characteristically paucibacillary, frequently resulting in negative acid-fast bacilli (AFB) smears and conventional cultures, which can misguide clinical teams and delay definitive care.

The clinical novelty of this case centers on its manifestation in an immunocompetent pediatric patient presenting with rapid, severe vision loss due to compressive optic neuropathy. It underscores the deceptive nature of the disease, where an initial negative AFB smear necessitated a high index of clinical suspicion and immediate endoscopic re-exploration to secure a diagnosis.

The objective of this case report is to describe a rare instance of isolated sphenoid sinus TB in a 10-year-old child presenting with profound vision loss. By analyzing this clinical course, we aim to highlight the complex diagnostic dilemmas associated with skull base EPTB, demonstrate the critical role of molecular assays like cartridge-based nucleic acid amplification testing (CBNAAT) in overcoming paucibacillary diagnostic failures, and emphasize the potential for complete functional visual restoration through prompt surgical decompression and targeted antitubercular therapy (ATT).

## Case presentation

A 10-year-old male child presented with complaints of a generalized headache and decreasing vision in his left eye for two months. This presentation was unaccompanied by fever, vomiting, or seizures. The patient’s vital signs were stable and within normal pediatric limits. On detailed neurological and ophthalmic examination, he was conscious and oriented, with all cranial nerves intact except for isolated left-sided optic nerve (cranial nerve II) dysfunction. Visual acuity in the right eye was normal (6/6), but the left eye was severely compromised, restricted to hand movements only. While the right pupil responded normally, the left pupil was sluggish, and a prominent relative afferent pupillary defect (RAPD) was confirmed during the swinging flashlight test. Fundoscopic evaluation revealed a normal right optic disc, whereas the left eye demonstrated distinct optic disc pallor and blurred nasal margins, consistent with compressive optic neuropathy. No other focal motor, sensory, or meningeal signs were present. The patient had an unremarkable medical background, with no history of previous hospitalizations or blood transfusions. His family history was negative for epilepsy, TB, asthma, or other chronic hereditary conditions. There was no known history of contact with TB. Born full-term via an uneventful lower segment cesarean section (LSCS) following a trial of vaginal delivery, he was appropriate for gestational age (AGA), cried immediately after birth (CIAB), and had no history of neonatal jaundice (NNJ) or neonatal intensive care unit (NICU) stay. Furthermore, his immunization coverage was fully up-to-date as per the National Immunization Schedule (NIS), and his developmental milestones were appropriately achieved for his age.

Baseline laboratory evaluations revealed marked neutrophilic leukocytosis (white blood cell (WBC) count: 32.33 × 10³/μL) and an elevated C-reactive protein level of 11.53 mg/L, while liver, kidney, and coagulation profiles were normal. Given the extensive osteolytic destruction of the clivus and sella turcica, a comprehensive endocrine panel was performed, confirming normal anterior pituitary functional integrity with preserved cortisol (30.5 μg/dL), prolactin (24.9 ng/mL), thyroid (thyroid-stimulating hormone (TSH): 0.48 μIU/mL, free thyroxine (FT4): 0.84 ng/dL), and gonadotropin levels. Screening for human immunodeficiency virus (HIV), hepatitis B surface antigen (HBsAg), and hepatitis C virus (HCV) and a Mantoux skin test were all negative. The aggressive bone erosion radiographically mimicked pediatric malignancies such as rhabdomyosarcoma or Ewing sarcoma or invasive fungal sinusitis.

MRI performed at an outside facility demonstrated a 33 x 29 x 35 mm mass lesion at the midline base of the skull with erosion of the sella turcica, extending into the pituitary fossa and bulging laterally into the cavernous sinus. A subsequent CT scan of the paranasal sinuses performed at our institute revealed a 3.4 x 2.9 x 3.2 cm lytic lesion involving the sphenoid sinus and the posterior ethmoidal air cells anteriorly (Figure 1). The lesion caused clear erosion of the clivus and the sphenoid sinus walls. Inferiorly, the mass was seen to bulge into the nasopharynx, while superiorly, it bulged into the anterior cranial fossa and the suprasellar cistern. A screening chest radiograph showed completely normal lung fields.

**Figure 1:**
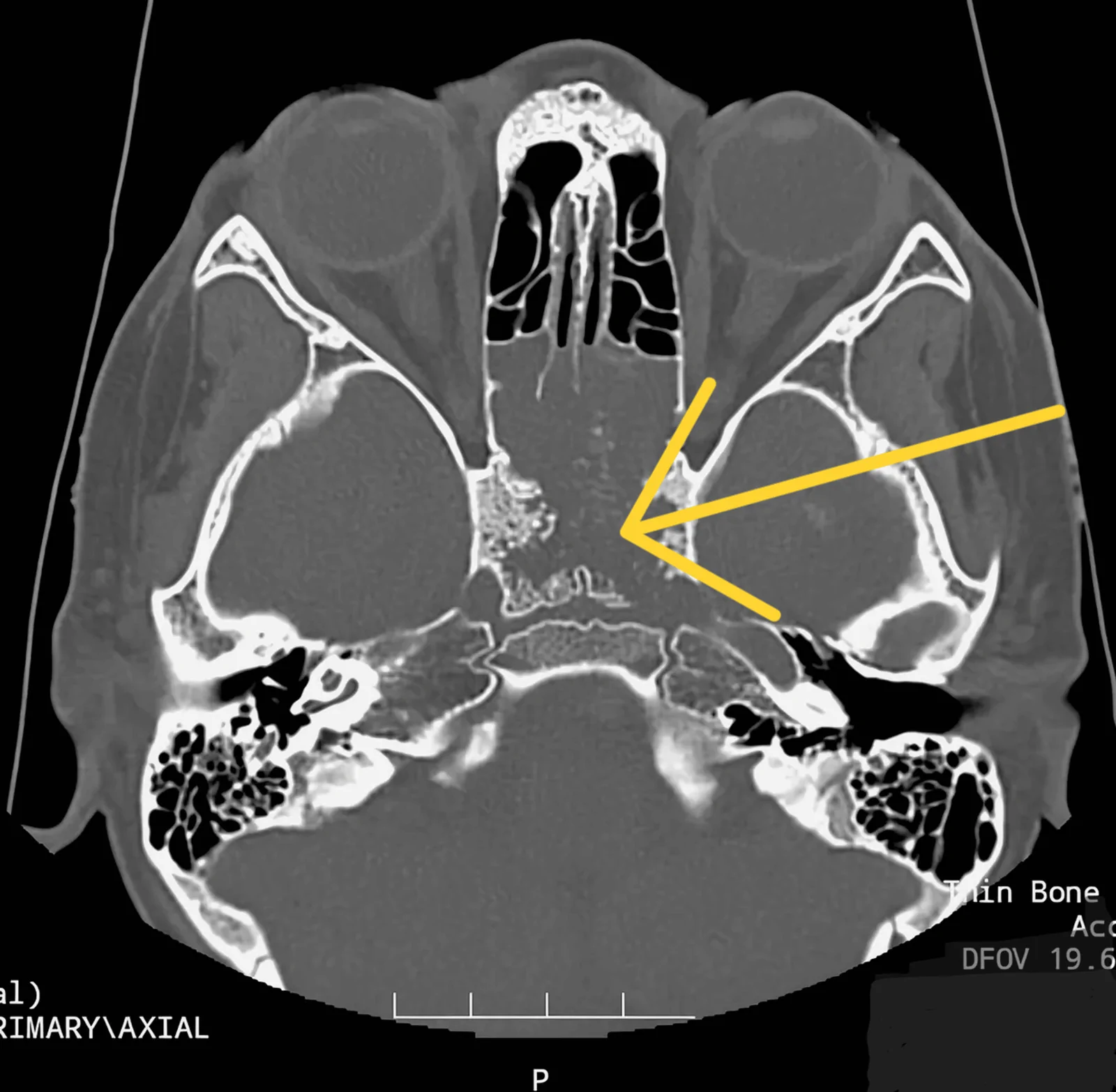
Preoperative Axial CT PNS (Bone Window) The scan demonstrates a large, expansile, 3.4 x 2.9 x 3.2 cm soft-tissue lytic lesion centered in the sphenoid sinus. Severe osteolytic bone destruction is visible along the sphenoid walls, extending posteriorly to erode the sella turcica and clivus, and anteriorly into the posterior ethmoid air cells. This aggressive pattern lacked classic infectious signs, radiographically masquerading as a skull base malignancy (e.g., rhabdomyosarcoma) or invasive fungal sinusitis. PNS: paranasal sinuses.

An endoscopic evaluation of the sphenoid sinus was carried out under general anesthesia. A partial middle turbinectomy was performed to gain access, and the sphenoethmoidal recess was approached. Soft, white, friable tissue was identified filling the sinus cavity (Figure 2). The tissue was surgically debulked and sent for histopathological examination. The initial biopsy revealed granulomatous inflammation with extensive necrosis and multinucleated giant cells, but no AFB were identified.

**Figure 2:**
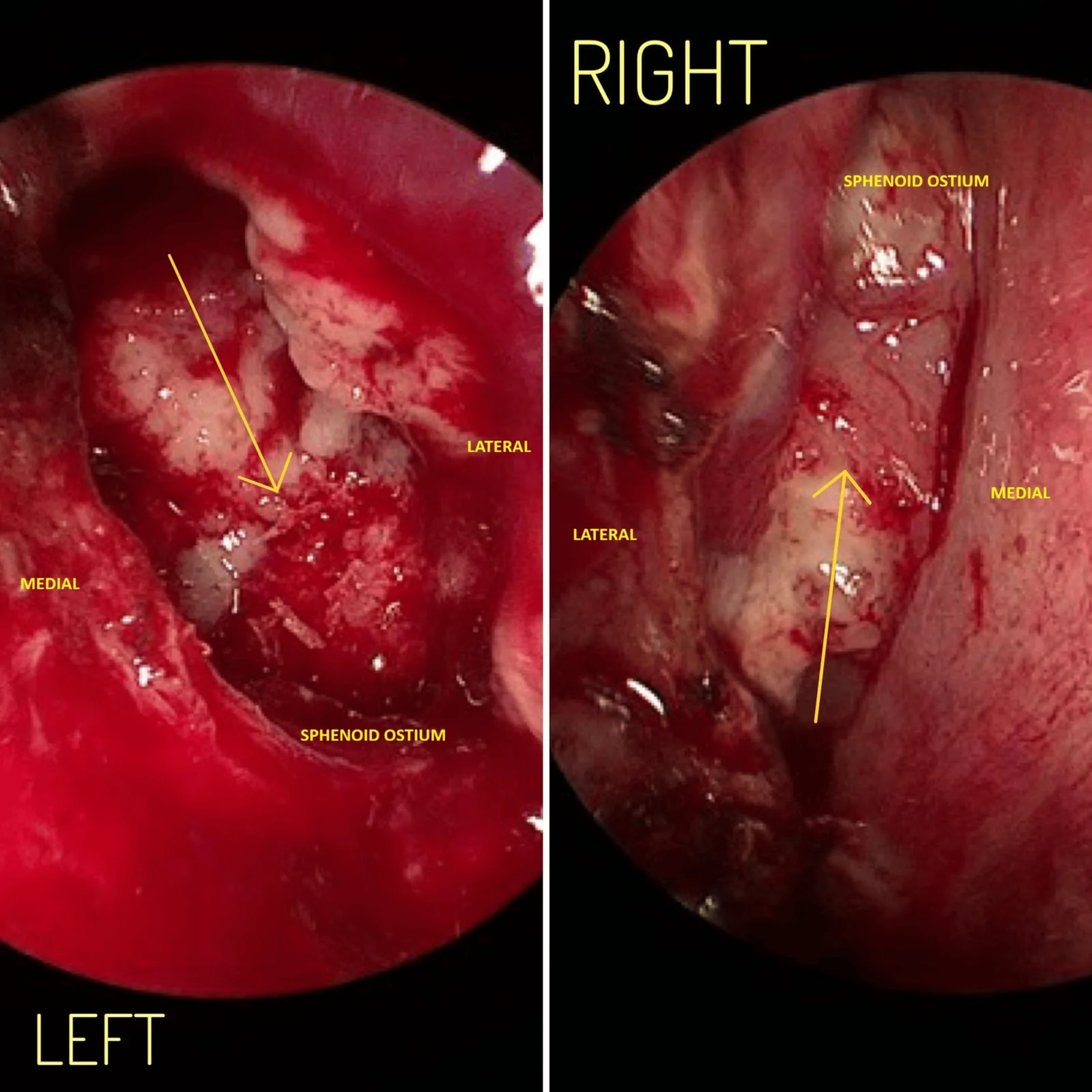
: Intraoperative Endoscopic Findings (Initial Surgery) Split-screen rigid endoscopic view of the sphenoid sinus. Arrows show soft, friable, white mass in the sphenoid ostium.

Postoperatively, the patient did not show immediate signs of clinical improvement, exhibiting a persistent headache and experiencing a single episode of a generalized seizure. Follow-up imaging indicated persistent disease burden. Due to the lack of clinical resolution, an endoscopic re-exploration was carried out. The cavities were inspected after further debulking (Figure 3). Tissue was re-harvested and sent for urgent biopsy, fungal culture and sensitivity testing, and CBNAAT. The CBNAAT assay returned positive for *Mycobacterium tuberculosis* and confirmed rifampicin sensitivity (no rifampicin resistance detected). 

**Figure 3:**
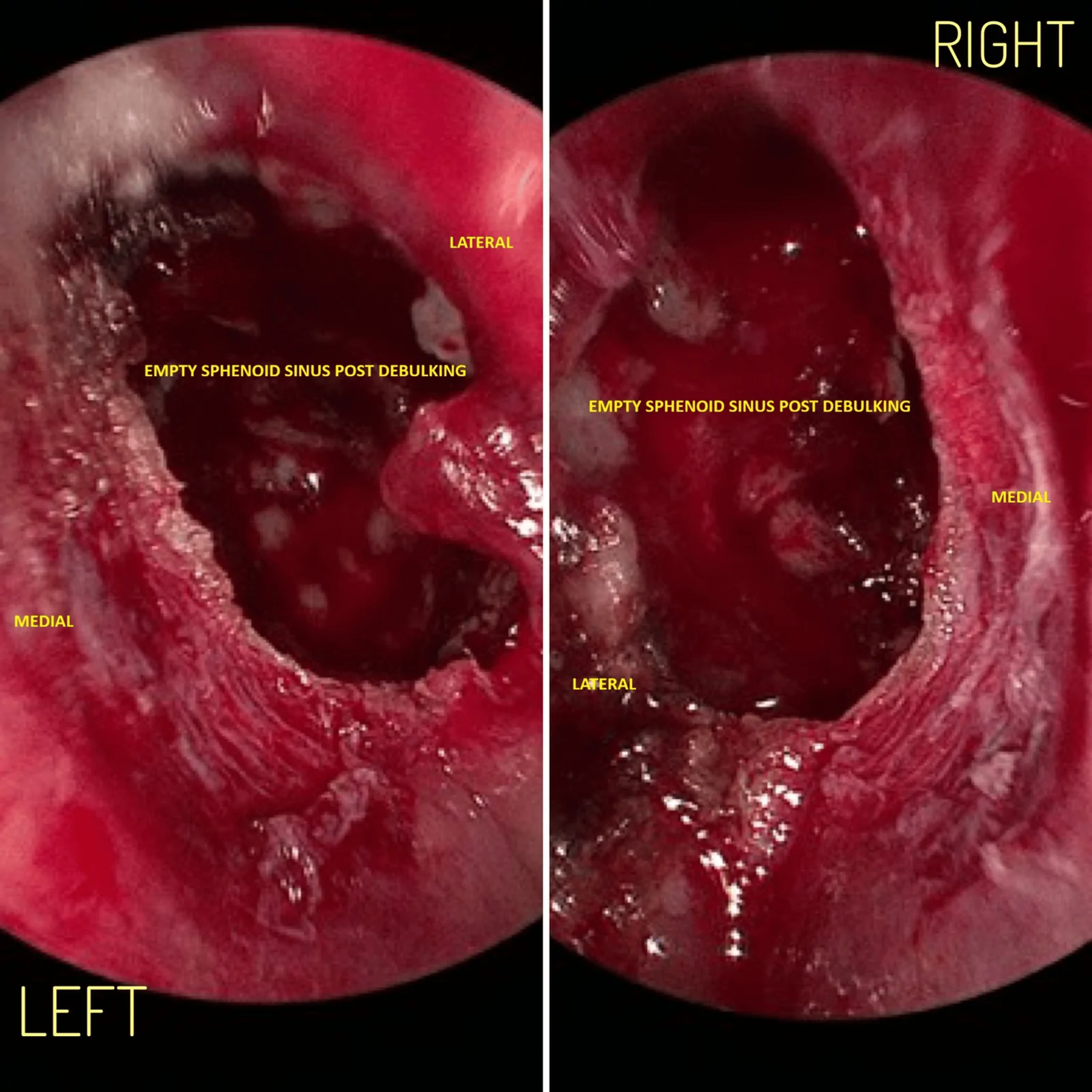
Endoscopic Findings During Re-exploration (Post-debulking) The image shows the sphenoid cavity after thorough repeat debridement of the friable mass, exposing the eroded walls and skull base defect. Deep tissue re-harvested from this posterior wall was sent for molecular testing, where CBNAAT definitively confirmed *Mycobacterium tuberculosis*.

Following molecular confirmation, the patient was initiated on a first-line pediatric four-drug ATT regimen consisting of rifampicin, isoniazid, pyrazinamide, and ethambutol (administered as Akurit-4). To manage acute complications and optimize recovery, several adjunctive therapies were implemented: intensive corticosteroid therapy using intravenous dexamethasone was given for four weeks to control intracranial inflammation and reduce compressive optic neuropathy before being transitioned to oral prednisolone and tapered over three weeks; antiepileptic coverage with levetiracetam was introduced and maintained following a postoperative generalized tonic-clonic seizure; and daily pyridoxine was co-prescribed alongside multivitamin supplements to prevent isoniazid-induced peripheral neurotoxicity. Additionally, hypertonic saline, mannitol, and acetazolamide were utilized short-term to manage transient cerebral edema and postsurgical CSF rhinorrhea, while oral nifedipine was temporarily required to control systemic hypertension secondary to Cushing's triad.

At one month of follow-up, the patient had no headache but showed no significant improvement in vision. At three months of follow-up, an ophthalmology consultation was performed, and the child had a visual acuity of 6/9. At six months of follow-up, visual acuity was found to be 6/6. The child continues to be monitored closely with regular follow-up. Table 1 shows the clinical timeline of the patient.

**Table 1:** Clinical Timeline of the Patient HM: hand movements; RAPD: relative afferent pupillary defect; CT: computed tomography; MRI: magnetic resonance imaging; AFB: acid-fast bacilli; CBNAAT: cartridge-based nucleic acid amplification test; ATT: antitubercular therapy.

Timeline	Clinical Status and Findings	Interventions and Management	Outcomes
Presentation (month 0)	Left eye vision loss (HM only) and headache for 2 months; Left RAPD and disc pallor	CT/MRI showing a 3.4 x 2.9 x 3.2 cm sphenoid mass eroding the sella/clivus	Malignancy or invasive fungal sinusitis suspected
First surgery	Soft, white, friable mass filling the sphenoid sinus	Endoscopic debridement and tissue biopsy	Pathology: Necrotic granulomatous inflammation; AFB negative
Post-op phase	Persistent headache, follow-up disease burden, and a single generalized seizure	Endoscopic re-exploration with repeat tissue harvesting	Tissue sent for urgent biopsy, fungal culture, and CBNAAT
Diagnosis	Confirmed tubercular etiology	CBNAAT positive for M. tuberculosis; initiated standard 4-drug ATT	Headache resolved; early clinical stabilization
1-month follow-up	Asymptomatic for headache	Continued ATT regimen	Left eye vision stable but no significant early change
3-month follow-up	Ophthalmology reassessment	Continued ATT regimen	Left eye vision significantly improved to 6/9
6-month follow-up	Final milestone evaluation	Completed standard ATT course	Complete functional recovery; left eye vision restored to 6/6

Table 2 shows the patient's visual acuity progression during inpatient and follow-up visits. 

**Table 2:** Visual Acuity Progression and Clinical Status During Follow-up

Follow-up Interval	Left Eye Visual Acuity	Clinical Status and Progression
Presentation (month 0)	Hand movements (HM) only	Severe visual impairment secondary to left cranial nerve II (optic nerve) compression.
1-month follow-up	No significant improvement	Patient reported complete resolution of headaches, but visual acuity remained impaired while continuing antitubercular therapy.
3-month follow-up	6/9	Demonstrated meaningful visual recovery during formal ophthalmology reassessment.
6-month follow-up	6/6	Achieved complete functional visual restoration following completion of therapy and ongoing close monitoring.

## Discussion

TB involving the paranasal sinuses is a rare manifestation of extrapulmonary TB, representing less than 1% of all head and neck TB presentations. Within this anatomical spectrum, the sphenoid sinus is the least commonly affected site, accounting for a mere 1% to 2% of sinonasal cases. This extreme rarity is primarily due to the deep-seated location of the sphenoid sinus at the central skull base, which shields it from direct exposure to inhaled droplets. Furthermore, its relatively poor vascularity and limited localized lymphatic drainage create an environment less hospitable to the survival and proliferation of *Mycobacterium tuberculosis*.

The pathogenesis of isolated sphenoid sinus TB can be categorized into primary and secondary mechanisms. Primary involvement occurs through the direct inhalation of airborne mycobacteria that bypass the anterior nasal filtration barrier to settle directly onto the sphenoid mucosal lining or through direct introduction via digital inoculation or instrumentation. The more common secondary involvement develops through three primary routes: hematogenous dissemination from a primary, often silent or dormant pulmonary focus; retrograde migration through deep cervical and skull base lymphatic chains; or direct contiguous extension from adjacent structures such as the nasopharynx, cribriform plate, clivus, or other paranasal sinuses.

Once lodged in the sinus mucosa, the bacilli trigger a classical cell-mediated hypersensitivity reaction, leading to the formation of granulomatous tissue. In a confined, rigid bony cavity like the sphenoid sinus, this expanding tissue leads to osteitis and subsequent bone resorption, as observed in this patient's clival and sellar destruction.

Radiological evaluation of sphenoid sinus TB is challenging due to the lack of pathognomonic features. On CT, the condition characteristically presents as an ill-defined soft tissue mass filling the sinus cavity, frequently accompanied by aggressive osteolytic destruction of the sinus walls, sella turcica, and clivus. Hyperdense foci or microcalcifications may be present within the soft tissue matrix, mimicking necrotic or mineralized tumor matrices. MRI typically shows T1-weighted hypointensity and T2-weighted variable signals with strong, heterogeneous postcontrast enhancement, indicating active inflammatory granulation. It also accurately delineates extension into the cavernous sinus or pituitary fossa.

Because these radiographic features are highly aggressive, they frequently lead to misdiagnoses of malignant or invasive skull base lesions. The primary differential diagnoses include malignancies such as small round blue cell tumors (e.g., rhabdomyosarcoma, Ewing sarcoma), chordomas, nasopharyngeal carcinoma, or lymphoma; benign extrasellar lesions like pituitary macroadenomas with suprasellar extension, craniopharyngiomas, or meningiomas; and invasive fungal sinusitis, such as mucormycosis or aspergillosis, which presents with similar bony erosion and neuro-ophthalmic deficits but requires a completely different therapeutic strategy.

Our patient, a 10-year-old immunocompetent male, presented with a progressive headache and visual loss. These are among the most common but nonspecific symptoms of sphenoid sinus pathologies. Such symptoms often delay diagnosis because they closely mimic more common conditions like chronic bacterial sinusitis, aggressive neoplasms, or invasive fungal infections [2,3]. In this case, initial imaging strongly suggested an aggressive, destructive skull base lesion eroding the sella and clivus with suprasellar extension, which initially raised a high index of suspicion for malignancy or invasive fungal disease.

Several cases sharing similarities with our patient have been reported in the medical literature. Sharma and Baruah described pediatric sphenoid sinus TB, noting that delayed diagnosis can directly lead to irreversible visual loss [4]. Turel and Rajshekhar reported a similar clinical presentation in an adolescent girl, also highlighting the masquerading nature of this disease as a skull base neoplasm that causes visual dysfunction [5]. More recently, Krishnamohan et al. documented a severe case leading to complete blindness and ophthalmoplegia, underscoring the stark consequences of delayed diagnosis [6]. Furthermore, literature reviews compiled by Bhat et al. reinforce that isolated sphenoid sinus lesions account for a tiny fraction of paranasal sinus pathologies, with a tubercular etiology in an inflammatory presentation frequently being an unexpected finding discovered after biopsy [7].

The standard management of this condition includes timely surgical intervention for diagnostic biopsy and decompression, particularly in cases demonstrating mass effect or obstructive neurological symptoms. This must be followed by a full course of ATT administered as per national guidelines, typically spanning six to nine months. Our patient showed excellent clinical improvement after surgical debridement and the targeted initiation of ATT, especially regarding his visual function. This serves as an important prognostic indicator showing that vision can be salvaged if definitive treatment is initiated before irreversible ischemic or compressive optic nerve damage occurs. This manuscript has been prepared and structured in accordance with the consensus-based CARE guidelines for clinical case reporting [8].

To combat diagnostic delays in paucibacillary cases, the World Health Organization strongly advocates for the use of rapid molecular diagnostics for TB detection [9]. Table 3 contrasts the clinical utility of standard diagnostic modalities. 

**Table 3:** Clinical utility of standard diagnostic modalities LJ: Löwenstein-Jensen medium; MGIT: Mycobacteria Growth Indicator Tube system; ATT: antitubercular therapy.

Diagnostic Modality	Sensitivity in Extrapulmonary Tissue	Turnaround Time	Key Limitations/Capabilities
Acid-fast bacilli (AFB) smear	Extremely low (<30%)	Hours	Failed to detect bacilli across multiple fields in both the first and second surgical biopsies.
Conventional mycobacterial culture (LJ/MGIT)	Low to moderate (40%–60%)	2 to 6 weeks	Delayed turnaround time limits its utility in acute compressive optic neuropathy, where immediate decompression is vital to save vision.
Cartridge-based nucleic acid amplification (CBNAAT / GeneXpert)	High (>85%–90%)	<2 hours	Definitively secured the diagnosis using deep sphenoid tissue and ruled out Rifampicin resistance, guiding immediate, targeted first-line ATT.

Table 4 shows the literature comparison of isolated sphenoid sinus TB. 

**Table 4:** Literature Comparison of Isolated Sphenoid Sinus Tuberculosis ATT: antitubercular therapy; HM: hand movements; AFB: acid-fast bacilli; CBNAAT: cartridge-based nucleic acid amplification test.

Study	Patient Demographics	Primary Clinical Presentation	Key Imaging and Pathological Findings	Interventions and Visual Outcome
Sharma and Baruah (2003) [4]	Pediatric	Progressive headache, visual loss	Isolated sphenoid lesion, delayed diagnosis	Surgical biopsy and ATT; highlighted risk of irreversible blindness
Turel and Rajshekhar (2013) [5]	Adolescent female	Visual dysfunction	Mimicked an aggressive skull base neoplasm	Surgical decompression followed by standard ATT course
Krishnamohan et al. (2024) [6]	Adult/adolescent	Complete blindness and ophthalmoplegia	Severe, advanced skull base erosion with cranial nerve palsies	Tardy diagnosis led to permanent, irreversible neuro-ophthalmic deficits
Bhat et al. (2020) [7]	Two-case series	Non-specific headache, unexpected post-biopsy finding	Minimal bone destruction; discovered accidentally via histopathology	Conservative surgical clearing and successful resolution with ATT
Current case (2026)	10-year-old immunocompetent male	Left vision loss (HM only) and headache; postoperative seizure.	34 x 29 x 32 cm mass eroding sella/clivus; initial biopsy AFB negative	Dual endoscopic surgeries, Tissue CBNAAT confirmation, 4-drug ATT + tapered steroids Complete visual restoration to 6/6

A high index of suspicion for TB must be maintained when encountering destructive skull base lesions that cause vision loss, even in pediatric patients with normal immune status and no history of TB contact. Because initial AFB smears are frequently negative in EPTB, clinicians should anticipate the potential need for repeat biopsies. To ensure accurate diagnosis in these paucibacillary cases, utilizing rapid molecular assays like CBNAAT is essential for both sensitive detection and resistance screening. Once diagnosed, prompt endoscopic decompression combined with early ATT is vital to salvage vision and prevent permanent neurological damage. Additionally, adjunctive treatments play a crucial role in patient management, with corticosteroids minimizing optic nerve edema and antiepileptics controlling secondary seizures.

## Conclusions

In the presented case of isolated sphenoid sinus TB, the condition masqueraded as an aggressive malignancy or invasive fungal infection, underscoring the diagnostic challenges associated with such elusive skull base lesions. The definitive diagnosis was established through the combined interpretation of clinical findings, advanced imaging, tissue pathology, and CBNAAT, rather than relying on a single diagnostic test. Within the limits of this specific case, the use of molecular assays on deep tissue specimens aided in overcoming initial paucibacillary diagnostic failures, while prompt surgical decompression and targeted ATT were associated with the patient's complete functional visual restoration.
